# Comparison of ChatGPT-Generated Patient Information on Testicular Cancer Across Two Assessment Periods: Clinical Accuracy, Response Quality, and Citation Support

**DOI:** 10.5152/tud.2026.26067

**Published:** 2026-11-02

**Authors:** Berk Yasin Ekenci, Mehmet Altan

**Affiliations:** 1 Department of Urology, Acıbadem Bayındır Söğütözü Hospital, Ankara, Türkiye; 2 Department of Urology, Etimesgut Şehit Sait Ertürk State Hospital, Ankara, Türkiye

**Keywords:** Artificial intelligence, information sources, large language models, patient education as topic, testicular neoplasms

## Abstract

**Objective:**

The aim of this study was to compare clinical accuracy, response quality, and citation characteristics of ChatGPT-generated testicular cancer information across 2 assessments 20 months apart.

**Methods:**

Sixty-one patient-oriented questions derived from European Association of Urology (EAU) Patient Information were submitted to ChatGPT-4o in December 2024 and repeated unchanged using ChatGPT-5.6 Luna in August 2026. Two urologists independently assessed accuracy against contemporaneous EAU guidelines and rated quality using the Global Quality Score (GQS) and modified DISCERN (mDISCERN). Citation quantity, source quality, and source-to-claim support were evaluated. Only the first response was retained; repeated-generation variability was not assessed.

**Results:**

Fully accurate responses were 86.9% (53/61) in 2024 and 93.4% (57/61) in 2026 (*P* = .125). Of 8 clinically relevant inaccuracies in 2024, 4 were corrected, 3 improved but remained misleading, and 1 persisted. GQS increased from 3.75 ± 0.77 to 4.82 ± 0.41 and mDISCERN from 30.37 ± 4.65 to 36.80 ± 1.93 (both *P* < .001). Citations per response decreased from 5.97 ± 3.53 to 4.79 ± 1.19 (*P* = .042), while high-quality unique sources increased from 77.4% to 96.9% (*P* < .001). Fully supported sources were 80.1% and 86.4% (*P* = .050); 6 sources (2.6%) in 2026 did not support the claims.

**Conclusion:**

Response quality and the use of high-quality sources were greater in 2026, whereas changes in clinical accuracy and source-to-claim support were less clear. Clinically meaningful errors persisted in guideline-sensitive topics. Because different model versions and interfaces were assessed, the findings represent model- and interface-specific snapshots, not a controlled head-to-head comparison.

**Main Points**

- Response quality scores were significantly higher in 2026 than in 2024, whereas the proportion of fully accurate responses did not differ significantly between assessments.
- Four of 8 clinically relevant inaccuracies identified in 2024 were corrected in 2026, whereas 4 remained clinically misleading.
- The proportion of high-quality sources increased markedly in 2026, despite fewer citations being provided per response.
- High-quality sources did not always support the claims for which they were cited, highlighting the need to evaluate source credibility and source-to-claim support separately.

## Introduction

Large language models (LLMs) are increasingly used for health information. Their value in patient education depends on clinical accuracy and reliability as well as fluent language. Patient-facing evaluations have reported unsafe responses, clinically relevant omissions, and guideline discordance.^1,2^ LLM-generated references may also be inaccurate, fabricated, or poorly matched to the claims they accompany.^1,3^ Source credibility and source-to-claim support are separate issues; an authoritative source may not substantiate the cited claim.^3^

LLM-generated patient information has been evaluated across several urologic subspecialties, with mixed results.^1,4-6^ Testicular cancer is a useful setting because it predominantly affects younger men and management depends on staging, treatment context, fertility, and survivorship.^7^ A recent testicular cancer study reported limited chatbot quality and actionability and reading levels above those recommended for patient education.^8^

Most evaluations are cross-sectional and assess a single model version.^1,5,6,8^ Because LLMs are updated frequently, findings from 1 time point may not represent later versions. Longitudinal studies have shown differences across successive ChatGPT versions, although changes vary by task.^9,10^ Repeating an unchanged question set reduces variation from question selection and study design. However, differences between assessments may also reflect changes in the deployed system, interface, or retrieval/citation behavior.

This study paired an unchanged 61-question testicular cancer set across 2 assessments approximately 20 months apart. We compared clinical accuracy against contemporaneous EAU recommendations, response quality and length, trajectories of 2024 inaccuracies, citation quantity, source quality, and source-to-claim support.

## Material and Methods

### Study Design and Ethical Considerations

Reporting followed the Chatbot Assessment Reporting Tool (CHART), developed for studies of generative artificial intelligence (AI) chatbots providing clinical evidence or health advice.^11^ A completed checklist is provided in Supplementary File 1.

This paired chatbot assessment used 2 predefined time points. The same 61 questions were submitted on December 22, 2024, and August 8, 2026, without changes in wording, order, or language; each question and its 2 responses formed a matched pair.

As the study involved no human participants, patient health information, biological specimens, or identifiable information, the authors considered institutional ethics committee review and informed consent unnecessary. No formal waiver or exemption was sought. Because no patient-level information was entered into ChatGPT, safeguards specific to patient health information were not applicable.

### Development of the Question Dataset

The question set was developed in 2024 from the content and topic structure of the EAU Patient Information on testicular cancer.^12^ Two urologists drafted 61 questions across 5 predefined sections: About the Testicles and Testicular Cancer (1-9), Risk Factors and Diagnosis (10-15), Treatments (16-41), Living with Testicular Cancer (42-58), and Recurrence (59-61). Questions were based on EAU topics rather than copied verbatim. No AI-assisted prompt optimization was performed after finalization, and no patients or public members were involved. The 2 urologists reviewed the set for clinical relevance, but no formal face or content validity assessment was performed. The complete questions are in Supplementary File 2. The 2026 assessment used the same 61 questions without modification; no a priori sample size calculation was performed.

### ChatGPT Query Procedure and Response Collection

The first assessment used ChatGPT-4o on December 22, 2024, and the second used ChatGPT-5.6 Luna on August 8, 2026 (OpenAI). Both used the publicly available ChatGPT web interface in Google Chrome incognito mode in Ankara, Türkiye, without an account. Each English-language question was entered in a new chat. The evaluated systems were closed-source proprietary models; no investigator-directed fine-tuning, retrieval augmentation, or parameter modification was performed. Release dates, development data, and technical parameters of the deployed builds were unavailable.

Both assessments used the same query procedure. Without an account, account-specific memory and custom instructions were unavailable. No browsing, search, or research mode was manually activated, although automatic retrieval or citation functionality could not be independently verified. Prompts were not modified, clarified, corrected, or regenerated; the first complete response was retained. After each primary response, the standardized follow-up “Can you provide references for this information?” was submitted. Citation analyses reflect elicited rather than spontaneous references. Each assessment comprised 61 primary and 61 follow-up prompts. Complete verbatim responses are provided in Supplementary Files 3 and 4.

### Reference Standard and Accuracy Assessment

Clinical accuracy was assessed against the EAU Guidelines on Testicular Cancer applicable at each time point. EAU Patient Information materials were used only to develop the question set, not as the accuracy reference standard. December 2024 responses were assessed against the 2024 EAU Guidelines on Testicular Cancer,^13^ and August 2026 responses against the 2026 edition.^7^ For questions involving fertility, hypogonadism, or testosterone replacement therapy, the corresponding EAU Guidelines on Sexual and Reproductive Health were also used.^14,15^ Responses were thus judged against the standard of care current at each assessment rather than against a single guideline version.

Each response was independently reviewed by 2 European Board of Urology–certified urologists. Reviewers were blinded to each other’s initial accuracy ratings but not to the assessment year or ChatGPT version. No patients or members of the public participated in the evaluation. Responses were classified as accurate, partially accurate but clinically misleading, or incorrect. Guideline-concordant stage-specific information without a material omission was considered accurate. Broadly correct information that overstated radiotherapy indications was considered partially accurate but clinically misleading, whereas material misclassification of disease stage was considered incorrect. An accurate response contained no clinically relevant statement inconsistent with contemporaneous EAU guidance and no material error that could alter patient understanding or management. A partially accurate but clinically misleading response was predominantly correct but contained a clinically relevant omission, overgeneralization, or inaccurate statement. An incorrect response contained a substantive error in diagnosis, staging, treatment, follow-up, or another clinically relevant area. Disagreements were resolved by joint reassessment against the relevant guideline until consensus; no third adjudicator was required.

Responses not fully accurate in 2024 were compared with their 2026 counterparts. Errors were classified as corrected, improved but persistent, or persistent according to whether the clinically relevant problem disappeared, improved but remained misleading, or remained substantially unchanged. Bias was not assessed separately.

### Response Quality Assessment

Response quality and reliability were independently assessed by the same 2 urologists using the Global Quality Score (GQS) and the same 8-item modified DISCERN (mDISCERN) instrument used in 2024. The 8-item mDISCERN was retained for comparability with the 2024 assessment and because its domains were applicable to the broader patient information content evaluated here; the original DISCERN places greater emphasis on treatment choices. GQS is a five-point ordinal scale of overall quality, flow, and usefulness, ranging from 1 (poor quality and of little use to patients) to 5 (excellent quality and highly useful).^16^ mDISCERN comprised 8 items: clarity of aims, achievement of aims, relevance, transparency of sources, currency, balance and absence of bias, provision of additional sources, and acknowledgment of uncertainty. Each item was scored from 1 to 5, for a total score of 8-40; higher scores indicated greater reliability and information quality.^17^ Reviewers were blinded to each other’s initial ratings but not to the assessment period or ChatGPT version. The full mDISCERN rubric is provided in Supplementary File 5.

### Citation and Source Assessment

For each response, total citations and unique sources were recorded separately; repeated citations to 1 source were counted once for source-level analyses. Unique sources were classified as high or low quality using predefined institutional and editorial criteria. High-quality sources included professionally governed guidelines, government agencies, peer-reviewed literature, academic centers, recognized cancer organizations, and evidence-based references. Low-quality sources lacked comparable transparent medical editorial oversight or evidence-based review. Commercial status alone did not determine classification. Full criteria are in Supplementary Table S1.

Source-to-claim support was classified as fully supported, partially supported, or unsupported by comparing each source with the claim(s) attributed to it. A source directly supporting the cited clinical statement was fully supported; one supporting only the broader topic or some claims was partially supported; one not substantiating the claim was unsupported. When a source supported multiple statements, classification reflected all claims and was conservative. Two reviewers assessed support independently and resolved disagreements by consensus. Web sources were judged from content available at the cited URL at review; archived versions were not systematically used.

### Response Characteristics

Response length was assessed by word count using the built-in Microsoft Word word count function. The same procedure was applied to both response sets, and word counts were compared between paired 2024 and 2026 responses.

### Statistical Analysis

Statistical analyses were performed using IBM SPSS Statistics, version 25.0 (IBM SPSS Corp.; Armonk, NY, USA). Gwet’s AC1 and its 95% confidence interval (CI) were calculated separately using R (version 4.6.1; R Foundation for Statistical Computing; Vienna, Austria) with the irrCAC package. Continuous variables are summarized as mean ± standard deviation (SD) or median and interquartile range (IQR), as appropriate; categorical variables are presented as frequencies and percentages. Normality of paired differences was assessed using the Shapiro–Wilk test. Response reproducibility across repeated generations was not evaluated because the predefined query protocol retained only the first complete response generated for each question at each assessment point.

Because the same 61 questions were evaluated in 2024 and 2026, paired statistical methods were used. For the primary clinical accuracy comparison, responses were dichotomized as fully accurate vs not fully accurate; the latter included partially accurate but clinically misleading and incorrect responses. The proportion of fully accurate responses was compared using the exact McNemar test. The original three-level accuracy classifications were also reported descriptively. Inter-rater agreement for the initial three-category ratings was summarized by exact agreement and Gwet’s AC1. Gwet’s AC1 was selected because marked imbalance among accuracy categories can make Cohen’s kappa unstable despite high observed agreement.

For GQS and mDISCERN, the mean of the 2 reviewers’ scores was calculated for each response. Paired GQS, mDISCERN, word counts, total citation counts, and unique-source counts were compared using the paired-samples *t* test when paired differences were normally distributed and the Wilcoxon signed-rank test otherwise. Inter-rater reliability for GQS and mDISCERN was assessed using the intraclass correlation coefficient (ICC) from a two-way mixed-effects model for absolute agreement based on the average of 2 raters (ICC[3,2]), with 95% CIs.^18^

For each response, the proportion of unique sources classified as high quality and the proportion classified as fully supported were calculated. All responses contained at least 1 cited source, so no response had a zero denominator in the response-level source-quality or source-to-claim support analyses. These paired response-level proportions were compared using the Wilcoxon signed-rank test because the paired differences were not normally distributed. Aggregate source counts and percentages were reported descriptively. Low-quality, partially supported, and unsupported sources were also summarized descriptively. All statistical tests were two-sided, and *P* value < .05 was considered statistically significant.

No formal adjustment for multiple comparisons was applied because clinical accuracy was designated as the primary analysis and the remaining comparisons were secondary and exploratory. Secondary *P*-values were interpreted cautiously.

### Use of AI

ChatGPT (GPT-5.6 Sol; OpenAI, San Francisco, CA, USA) was used during manuscript preparation for English-language editing, grammar correction, stylistic refinement, and assistance with phrasing and organization of author-written text. It was not used to independently perform statistical analyses, generate study data, assign clinical accuracy categories, score GQS or mDISCERN, or classify source quality or source-to-claim support. The authors reviewed and verified all AI-assisted suggestions, made all final scientific interpretations and decisions, and take full responsibility for the manuscript. This writing assistance use was separate from the use of ChatGPT-4o and ChatGPT-5.6 Luna as the systems evaluated under the predefined study protocol.

## Results

The proportion of fully accurate responses was 86.9% (53/61) in 2024 and 93.4% (57/61) in 2026, with no statistically significant difference between assessments (*P* = .125). Four responses that were not fully accurate in 2024 became fully accurate in 2026, while no response classified as fully accurate in 2024 deteriorated. Among the 8 responses containing clinically relevant inaccuracies in 2024, 4 were fully corrected in 2026 (questions 19, 27, 54, and 61). Three improved but remained partially accurate and clinically misleading (questions 8, 9, and 21), and 1 remained partially accurate and clinically misleading (question 26). The status, clinical relevance, and representative 2024 and 2026 content of these responses are detailed in Table 1.

**Table 1.** Reassessment in 2026 of Clinically Relevant Inaccuracies Identified in the 2024 Assessment

Question No.	Clinical Topic	2024 Assessment	Representative Incorrect or Misleading Content	Potential Patient Misunderstanding or Clinical Consequence	2026 Reassessment and Representative Content
8	Disease staging	Incorrect	Stage III was described as requiring distant spread with substantially elevated tumor markers, omitting stage groups defined by regional nodal disease with elevated post-orchiectomy serum markers.	Could lead to incorrect stage assignment and misunderstanding of prognosis or stage-specific management.	Improved but persistent: the 2026 response better incorporated metastatic status and serum tumor markers, but stage III grouping remained simplified and did not fully account for regional nodal disease with S2 and S3 marker levels.
9	TNM staging	Incorrect	Stage IIIB/IIIC grouping was inaccurate, particularly in the integration of post-orchiectomy serum marker categories with metastatic status.	Could lead to incorrect understanding of TNM stage assignment and its implications for prognosis and management.	Improved but persistent: post-orchiectomy serum markers were incorporated into staging, but overall stage grouping remained simplified.
19	Testicular prosthesis	Partially accurate but clinically misleading	Rare complications of testicular prosthesis implantation were presented as routine postoperative risks.	Could overstate the expected risk of prosthesis-related complications and discourage consideration of an otherwise reasonable option.	Corrected: complications were presented with greater qualification, including recognition that several events are uncommon or rare.
21	Stage-specific treatment	Incorrect	Stage- and histology-specific treatment pathways were insufficiently differentiated.	Could create incorrect expectations regarding stage-appropriate treatment and the need for additional therapy.	Improved but persistent: treatment was more clearly differentiated by stage and histology, but some stage-specific recommendations remained overgeneralized.
26	RT	Partially accurate but clinically misleading	The role of RT was presented more broadly than supported by contemporary guideline recommendations.	Could lead patients to believe that RT is indicated more broadly than recommended by contemporary guidelines.	Persistent: RT was described as mainly relevant to seminoma and increasingly selective, but its role remained broader than contemporary guideline recommendations.
27	RT adverse effects	Partially accurate but clinically misleading	Potential RT adverse effects were presented without adequate context regarding current indications and expected frequency.	Could exaggerate perceived treatment toxicity and cause unnecessary concern regarding RT-related adverse effects.	Corrected: adverse effects were presented in the context of selected seminoma treatment and with greater qualification regarding frequency and treatment field.
54	TRT	Incorrect	Relative contraindications to testosterone replacement therapy were presented as absolute.	Could lead patients to interpret relative contraindications to testosterone replacement therapy as absolute and misunderstand treatment eligibility.	Corrected: TRT suitability was presented as requiring individualized assessment rather than treating relative contraindications as uniformly absolute.
61	Recurrent disease	Partially accurate but clinically misleading	Non-standard treatment approaches were presented alongside established salvage treatment options for recurrent disease.	Could confuse established salvage treatment strategies with non-standard approaches and distort expectations regarding management of recurrent disease.	Corrected: recurrent disease management was organized around established salvage chemotherapy, selected surgery, high-dose chemotherapy with stem cell support, and prior-treatment context.

RT, radiotherapy; TRT, testosterone replacement therapy; TNM, tumor–node–metastasis.

Exact inter-rater agreement for the initial clinical accuracy classification was 96.7% (59/61) in 2024 and 95.1% (58/61) in 2026. Gwet’s AC1 was 0.963 (95% CI: 0.91-1.00) and 0.950 (95% CI: 0.89-1.00), respectively. Initial disagreement occurred in 2 of 61 responses (3.3%) in 2024 and 3 of 61 responses (4.9%) in 2026. All disagreements were resolved by joint reassessment against the relevant contemporaneous EAU guideline; no third adjudicator was required.

Response quality scores were higher in 2026 than in 2024. The mean GQS increased from 3.75 ± 0.77 to 4.82 ± 0.41 (*P* < .001), and the mean mDISCERN increased from 30.37 ± 4.65 to 36.80 ± 1.93 (*P* < .001). Inter-rater reliability was high for both instruments at both assessment points. The ICCs for GQS were 0.944 (95% CI: 0.907-0.967) in 2024 and 0.902 (95% CI: 0.836-0.941) in 2026; corresponding ICCs for mDISCERN were 0.960 (95% CI: 0.932-0.976) and 0.891 (95% CI: 0.818-0.935). The mean response length increased from 304.52 ± 92.30 to 340.33 ± 113.31 words (*P* = .001). Descriptive domain-level analyses showed higher mean GQS and mDISCERN scores in 2026 across all 5 predefined question domains (Supplementary Table S2). Clinical accuracy and response quality comparisons are summarized in Table 2.

**Table 2.** Comparison of Clinical Accuracy and Response Quality Between the 2024 and 2026 Assessments

Outcome	2024	2026	Paired Effect Estimate (95% CI)	*P*
Fully accurate, n (%)	53 (86.9)	57 (93.4)	6.6 pp (−1.5 to 15.9)	.125
GQS, mean ± SD; median (IQR)	3.75 ± 0.77; 4.00 (3.00-4.00)	4.82 ± 0.41; 5.00 (5.00-5.00)	1.0 (0.8-1.3)	<.001
mDISCERN, mean ± SD; median (IQR)	30.37 ± 4.65; 31.00 (27.75-33.50)	36.80 ± 1.93; 37.00 (36.00-38.00)	6.5 (5.5-7.5)	<.001
Word count, mean ± SD	304.52 ± 92.30	340.33 ± 113.31	35.8 (15.1-56.5)	.001

Data are presented as mean ± SD, median (IQR), or n (%), as appropriate. Paired effect estimates are expressed as 2026 minus 2024. The clinical accuracy comparison was performed using the exact McNemar test, and the effect estimate represents the absolute paired difference in the proportion of fully accurate responses. The mean paired differences with 95% CIs are reported for outcomes analyzed using paired-samples *t* tests, whereas Hodges–Lehmann paired estimates with 95% CIs are reported for outcomes analyzed using Wilcoxon signed-rank tests. CIs for secondary outcomes are unadjusted for multiplicity. CI, confidence interval; GQS, Global Quality Score; IQR, interquartile range; mDISCERN, modified DISCERN; pp, percentage points; SD, standard deviation.

The total number of citations decreased from 364 in 2024 to 292 in 2026, and citations per response decreased from 5.97 ± 3.53 to 4.79 ± 1.19 (*P* = .042). The number of unique sources decreased from 261 to 228, but the mean number of unique sources per response did not differ significantly (4.28 ± 2.01 vs 3.74 ± 1.37; *P* = .101).

The proportion of high-quality unique sources increased from 77.4% (202/261) in 2024 to 96.9% (221/228) in 2026 (*P* < .001). Fully supported unique sources increased from 80.1% (209/261) to 86.4% (197/228), but the paired comparison was not statistically significant (*P* = .050). Partially supported unique sources decreased from 19.9% (52/261) to 11.0% (25/228), while 6 unsupported unique sources were identified in 2026 (2.6%) and none in 2024. Unsupported sources occurred in responses to questions 19, 26, 28, 33, 36, and 45. One representative example was a study comparing 2 adjuvant radiotherapy doses that was cited as evidence for a radiotherapy-versus-surveillance comparison. Another was a US Food and Drug Administration resource on reproductive-tissue regulation and donor screening that was cited to support statements about where sperm-bank and fertility services could be accessed. No cited reference was inaccessible or broken, no paywall prevented verification, and no bibliographically fabricated reference was identified. Detailed comparisons of citation quantity, source quality, and source-to-claim support are presented in Table 3.

**Table 3.** Comparison of Citation Characteristics Between the 2024 and 2026 Assessments

Outcome	2024	2026	Paired Effect Estimate (95% CI)	*P*
Total citations, n	364	292	—	—
Citations per response, mean ± SD; median (IQR)	5.97 ± 3.53; 5 (4-6.5)	4.79 ± 1.19; 5 (4-5)	−1.0 (−2.1 to −0.2)	.042
Unique sources, n	261	228	—	—
Unique sources per response, mean ± SD	4.28 ± 2.01	3.74 ± 1.37	−0.54 (−1.19 to 0.11)	.101
Source Quality, n (%)				
High	202 (77.4)	221 (96.9)	20.0 pp (13.0-25.0)	<.001
Low	59 (22.6)	7 (3.1)	—	
Source-to-Claim Support, n (%)				
Fully supported unique sources	209 (80.1)	197 (86.4)	7.0 pp (0.3-17.2)	.050
Not fully supported unique sources	52 (19.9)	31 (13.6)	—	
Partially supported unique sources	52 (19.9)	25 (11.0)	—	—
Unsupported unique sources	0 (0)	6 (2.6)	—	—

Data are presented as mean ± SD, median (IQR), or n (%), as appropriate. Paired effect estimates are expressed as 2026 minus 2024. Mean paired differences with 95% CIs are reported for outcomes analyzed using paired-samples t tests, whereas Hodges–Lehmann paired estimates with 95% CIs are reported for outcomes analyzed using Wilcoxon signed-rank tests. For source quality and source-to-claim support, effect estimates and *P* values are based on paired response-level proportions of unique sources classified as high quality and fully supported, respectively; aggregate source counts and percentages are presented descriptively. Total citation counts, total unique-source counts, and complementary source categories were not tested separately. CIs for secondary outcomes are unadjusted for multiplicity. CI, confidence interval; IQR, interquartile range; pp, percentage points; SD, standard deviation.

On the primary dichotomized accuracy outcome, 4 of 61 responses (6.6%) improved from not fully accurate to fully accurate, none worsened, and 57 of 61 (93.4%) remained unchanged. Using the original three-category classification, 7 responses improved by at least 1 category, none worsened, and 54 remained unchanged. The exact paired transition matrix is provided in Supplementary Table S3.

## Discussion

Across the 2 assessment periods, ChatGPT-generated patient information differed in several respects. Fully accurate responses were numerically more common in 2026 than in 2024 (93.4% vs 86.9%), but the paired difference was not statistically significant. Four of 8 responses with clinically relevant inaccuracies in 2024 became fully accurate, while 4 remained clinically misleading in 2026. GQS and mDISCERN scores were higher in 2026. Citation counts decreased, but a larger proportion of sources were of high quality; the change in fully supported sources was smaller and not statistically significant. Improvements were clearest for response quality and source quality, not for clinical accuracy or source-to-claim support.

Differences between successive LLM versions have also been reported in other medical settings. Using the same 120 anatomy questions over approximately 2 years, Paslı and Günenç Beşer observed an accuracy of 45.8% with ChatGPT-3.5, 73.3% with ChatGPT-4o, and 86.7% with ChatGPT-5.^9^ A longitudinal clinical neurology study similarly reported higher accuracy and comprehensiveness with GPT-5.2 than with GPT-3.5, with variation across subspecialties.^19^ Version-dependent differences have also been reported in urology.^20,21^ Unlike examination-based studies or cross-sectional model comparisons, the present study reassessed the same patient-oriented questions against EAU recommendations current at each time point.

The remaining errors clustered in questions that required context-sensitive interpretation of stage, histology, treatment setting, or guideline recommendations rather than simple factual recall. Small differences in stage, post-orchiectomy tumor marker status, histology, and prior treatment can change testicular cancer management. Seminoma and nonseminoma differ in the role and timing of chemotherapy, radiotherapy, and surgery, and radiotherapy is appropriate only in selected stage- and histology-specific settings.^7^ Fertility preservation may need to be addressed before potentially gonadotoxic treatment, while recurrent-disease management depends on prior therapy and the salvage setting.^7,15^ A response can be broadly correct yet clinically misleading if these qualifiers are omitted or overgeneralized. Similar context-related limitations have been reported in urological evaluations of ChatGPT.^1,22^ Other studies have found good performance for many routine questions on lower urinary tract symptoms suggestive of benign prostatic enlargement and endourology.^4,23^ In our data, general patient information was usually accurate, whereas questions requiring clinical interpretation remained more vulnerable to error.

The improvement in GQS and mDISCERN was much larger than the change in binary accuracy. These instruments assess organization, completeness, usefulness, and reliability rather than guideline concordance alone. The 2026 GQS distribution was concentrated at the top of the scale (median: 5.00; IQR: 5.00-5.00), suggesting a ceiling effect that may have reduced discrimination among already high-scoring responses. Previous urological studies have likewise found that high quality or DISCERN scores do not necessarily imply high actionability, readability, or clinical completeness.^5,6,8^ We did not assess these domains separately. Differences in models, prompts, question selection, and instruments also limit direct comparisons across studies. In our data, better organization and completeness did not always correspond to greater clinical accuracy. Higher GQS and mDISCERN scores should not be interpreted as evidence of improved clinical safety. Longer responses in 2026 may also have increased perceived completeness and organization without improving accuracy.

ChatGPT produced fewer citations in 2026, but a larger proportion came from high-quality sources. Whiles et al found citation problems in 92.3% of responses when ChatGPT was specifically asked for sources.^1^ More recent work by Lin et al reported greater citation validity with ChatGPT-4 than with ChatGPT-3.5.^21^ In our study, fewer citations in 2026 were accompanied by greater use of authoritative sources.

Source quality did not ensure source-to-claim support. Fully supported unique sources increased from 80.1% to 86.4%, but the paired difference was not statistically significant, and 6 unsupported unique sources were identified in 2026. This matters clinically because an authoritative citation can increase confidence in a statement even when it does not substantiate that statement. In patient-facing information, such mismatches may create false reassurance, obscure uncertainty, or make inadequately supported claims appear externally validated. Source credibility should not be treated as evidence of claim-level support. Cheng et al reported a similar discrepancy in AI-generated scientific writing: 60.4% of references were accurate, but only 22.2% of citations were highly relevant to the associated statements.^3^ A recent evaluation of a ChatGPT-5-generated review on penile prosthesis implantation also found good factual accuracy with weaknesses in citation reliability and evidence synthesis.^24^ Citation assessment should distinguish source credibility from support for the specific claim. Thus, better source quality did not translate into a statistically significant improvement in source-to-claim support.

A strength of the design is the paired use of an unchanged question set with contemporaneous guideline standards and separate assessment of source quality and source-to-claim support. This study has several limitations. First, ChatGPT-4o was evaluated in 2024 and ChatGPT-5.6 Luna in 2026. The observed differences cannot be attributed to temporal change alone or interpreted as a controlled head-to-head comparison. Default web interface settings and automatic retrieval or citation behavior may also have differed, although no browsing, search, or research mode was manually activated. References were elicited with a standardized follow-up prompt rather than generated spontaneously in the initial response, and model-specific behavior in response to this prompt may have affected citation findings. Only the first response to each question was analyzed, so repeated-generation variability was not assessed.

The EAU-derived questions were more structured than many real-world patient prompts and may underestimate difficulty with incomplete or ambiguous queries.^2^ Reviewers knew the assessment period and ChatGPT version, which may have introduced expectation bias, especially for subjective quality ratings. GQS and mDISCERN may favor well-organized and complete responses without directly measuring clinical accuracy. Dedicated readability and actionability instruments were not included.

Web-based sources were assessed from content available at the time of review rather than from systematically archived versions, so later changes to online content cannot be excluded. Support was assigned at the unique-source level across all claims for which a source was used; a broadly relevant source could be rated only partially supported if it failed to substantiate one or more specific claims. All prompts and responses were in English, limiting generalizability to other languages. Potential harms were described qualitatively, but no predefined or validated safety-severity scale was used. The study evaluated response content rather than patient comprehension, trust, interpretation, or behavior. Source-quality classification also relied on predefined institutional and editorial criteria rather than a validated rating instrument. Finally, the findings apply only to the ChatGPT versions and interface conditions evaluated here and should not be generalized to other LLMs or future releases.

Compared with the 2024 assessment, ChatGPT-generated testicular cancer information showed higher response quality and a greater proportion of high-quality sources in 2026, whereas the change in clinical accuracy was not statistically significant and source-to-claim support remained imperfect. Clinically meaningful errors persisted in guideline-sensitive areas, including staging and treatment-related questions. ChatGPT may be useful as an adjunct for general patient education, but responses concerning staging, treatment selection, recurrence, fertility preservation, or hormonal therapy should be checked against current professional guidelines. Urologist oversight remains necessary, and cited sources should be verified for support of the specific claims for which they are used.

## Data Availability

The complete study question set and verbatim ChatGPT responses from both assessment periods are provided in Supplementary Files 2-4. Additional data underlying the reported evaluations are available from the corresponding author upon reasonable request. No custom model or model parameters were developed as part of this study, and the technical parameters of the proprietary ChatGPT models were not accessible to the investigators.
